# FAP-targeted [^68^Ga]BED003-PET compared with [^18^F]DOPA-PET in patients with recurrent medullary thyroid carcinoma

**DOI:** 10.3389/fnume.2026.1949036

**Published:** 2026-10-21

**Authors:** David Ventura, Benjamin Noto, Philipp Backhaus, Philipp Schindler, Kambiz Rahbar, Michael Schäfers, Burkhard Riemann, Wolfgang Roll

**Affiliations:** 1 Department of Nuclear Medicine, University Hospital Münster, Münster, Germany; 2 West German Cancer Center, University Hospital Münster, Münster, Germany; 3 European Institute for Molecular Imaging (EIMI), University of Münster, Münster, Germany; 4 Clinic for Radiology, University Hospital Münster, Münster, Germany

**Keywords:** BED003, DOPA, FAPI-PET, MTC, OncoFAP, thyroid cancer

## Abstract

**Aim:**

Molecular imaging of recurrent or advanced medullary thyroid carcinoma (MTC) remains clinically challenging. [^18^F]DOPA-PET is an established reference standard, but its sensitivity is limited and dependent on tumour burden and biomarker levels. Fibroblast activation protein (FAP)-targeted PET has emerged as a promising imaging approach across several malignancies. This study aimed to compare the detection rate and quantitative imaging characteristics of [^68^Ga]BED003-PET, recently known as [^68^Ga]OncoFAP-DOTAGA, with [^18^F]DOPA-PET in patients with recurrent or advanced MTC.

**Methods:**

Eight patients with histologically proven MTC and suspected recurrence or progression based on elevated calcitonin levels underwent both [^18^F]DOPA-PET and an additional [^68^Ga]BED003-PET. All patients presented with inconclusive findings in [^18^F]DOPA-PET. Comparative analyses were performed on a patient- and lesion-based level. Quantitative parameters included maximum and mean standardized uptake values (SUV_max_/SUV_mean_), metabolic tumour volume (MTV), and maximum tumour-to-background ratios (TBR_max_). Potential theranostic suitability was explored based on lesional FAP uptake.

**Results:**

The median interval between PET acquisitions was 33 days (range, 7–56 days). In total, 35 tracer-avid lesions were detected. [^68^Ga]BED003-PET detected significantly more lesions than [^18^F]DOPA-PET (34/35 [97%] vs. 16/35 [46%], *P* < 0.001). 15 lesions (43%) were positive on both modalities, 19 (54%) were only [^68^Ga]BED003-positive, and 1 (2.9%) was only [^18^F]DOPA-positive. Quantitative analysis demonstrated significantly higher uptake and contrast for [^68^Ga]BED003, including higher blood-pool TBRmax than [^18^F]DOPA (6.02 vs. 2.23, Bonferroni adjusted *P* < 0.001).

**Conclusion:**

In this small MTC cohort, [^68^Ga]BED003-PET demonstrated higher lesion detection and TBRs in patients with inconsistent findings in [^18^F]DOPA-PET. Prospective multicenter validation is warranted.

## Introduction

Medullary thyroid carcinoma (MTC) is a rare neuroendocrine malignancy arising from calcitonin-secreting parafollicular thyroid C-cells and accounts for approximately 1–2% of thyroid cancers (1). Surgery remains the only potentially curative treatment, whereas recurrent or metastatic disease is managed with local therapies in selected cases and systemic treatment in progressive disease (2). During follow-up, calcitonin and carcinoembryonic antigen are established tumour markers, and molecular imaging is essential for localizing disease and guiding treatment decisions. Conventional imaging, including neck ultrasound, CT, and MRI, remains an important component of the diagnostic work-up and follow-up of patients with MTC (2, 3).

Among PET tracers, [^18^F]DOPA is an established reference for imaging recurrent MTC, although detection is influenced by calcitonin levels and disease burden (4–7). However, its clinical availability remains limited to relatively few specialized centres (2). Somatostatin receptor (SSTR)-directed PET is increasingly used as a complementary approach, also identifying candidates for potential peptide receptor radionuclide therapy (PRRT) (4). [^18^F]fluorodeoxyglucose (FDG)-PET may provide additional diagnostic value, particularly in patients with more aggressive or poorly differentiated disease (8). However, comparative studies indicate that [^18^F]DOPA generally shows superior lesion detection compared with both SSTR-PET and [^18^F]FDG-PET, although these tracers may provide complementary information in selected patients (9–12).

Fibroblast activation protein (FAP)-targeted PET has emerged as a promising imaging approach in oncology (13). In MTC, stromal FAP expression provides a biological rationale for FAP-directed imaging (14). Initial clinical data suggested superior detection of distant metastases with FAPI-PET compared with SSTR-PET (15, 16), and a systematic review reported high detection rates for FAP-guided PET in thyroid cancer while emphasizing the limited evidence (17). More recently, a retrospective head-to-head study comparing [^68^Ga]FAPI-PET with [^18^F]DOPA-PET in recurrent MTC reported a higher number of detected metastases and higher tumour-to-background ratios (TBR) for [^68^Ga]FAPI-PET, supporting further evaluation of this target in MTC (18).

[^68^Ga]BED003 is a FAP-targeting tracer derived from the OncoFAP platform (19, 20). OncoFAP was developed as an ultra-high-affinity small-molecule FAP ligand and subsequently translated into clinical PET imaging, demonstrating high uptake across different solid malignancies (21).

The aim of this study was to compare lesion detection and quantitative imaging characteristics of [^68^Ga]BED003-PET and [^18^F]DOPA-PET in patients with MTC and to discuss potential theranostic implications.

## Methods

### Patients’ characteristics

This is a retrospective single-center study. Patients with suspected recurrent and/or metastasized MTC that had [^18^F]DOPA- and [^68^Ga]BED003-PET fewer than 60 days apart between 12/23 and 12/24 were included. All patients received [^18^F]DOPA-PET prior to [^68^Ga]BED003-PET. In all patients, elevated and/or progressed calcitonin levels were observed, which did not correspond with the imaging results after [^18^F]DOPA. This finding underscores the necessity for further imaging procedures to be conducted. Patients were subjected to an additional [^68^Ga]BED003-PET examination on the grounds of the encouraging detection rates reported in the literature in comparison to SSTR-PET (15). There were no general exclusion criteria for imaging with [^68^Ga]BED003. As retrospectively assessed all patients imaged with PET between 12/23 and 12/24 received both [^18^F]DOPA- and [^68^Ga]BED003-PET. Patient #1, #3, #5 and #6 were reported (not in detail, only a maximum intensity projection (MIP) of one patient and reference SUV_max_) recently in our multicancer analysis of [^68^Ga]BED003 (21). This analysis was approved by the ethics committee of the Westphalia-Lippe and the medical faculty of the University of Münster (approval no. 2025–388-f-S). Detailed patients’ characteristics are presented in Table 1.

**Table 1:** Patients’ characteristics.

Characteristics	N	%	median	Range
Patients	8			
Male	3	38		
Female	5	62		
Age [years]			60	32–78
MEN2^a^-Syndrome	1/8	13		
Pretreatments				
Thyroidectomy	8/8	100		
Cervical lymphadenectomy	8/8	100		
Surgery of distant metastasis	4/8	50		
Radiation therapy	1/8	13		
Patient-based findings				
Local recurrence	1/8	13		
Lymph node metastasis	6/8	75		
Distant metastasis	5/8	63		
Bone	3/8	38		
Lung	2/8	25		
Liver	2/8	25		
Tumor marker at the timepoint of [^18^F]DOPA-PET				
Calcitonin in pg/mL			1631.5	128–33981
CEA* in ng/mL			40.7	3.4–764

a MEN2-syndrome, multiple endocrine neoplasia 2 syndrome; CEA, carcinoembryonic antigen.

### [^18^F]DOPA-PET and [^68^Ga]BED003-PET

[^18^F]DOPA and [^68^Ga]BED003 were produced in-house in our radiochemistry unit based on clinical standards as described previously (20, 22). [^18^F]DOPA-PET was acquired 30-60 minutes after the intravenous administration of 2 MBq/kg, in accordance with recommendations from previous oncological studies (23). [^68^Ga]BED003-PET was acquired 60 ± 10 minutes after the intravenous administration of 2.5 MBq/kg, based on our previously reported pharmacokinetic studies (20). Images were acquired using either a Biograph 128 mCT system (acquisition speed of 1.1 mm/s) or a 3 T Biograph mMR system (acquisition time of 3 minutes per bed position; Siemens Healthcare). In regard to PET with computed tomography (CT), a supplementary low-dose CT scan of the PET volume was conducted for the purpose of attenuation correction. Additional CT was acquired based on individual clinical settings with non-ionic iodinated contrast agent (Ultravist, Bayer, Germany). In regard to PET with magnetic resonance imaging (MRI) additional axial T1- and T2 weighted and axial/coronal T1-weighted fat-suppressed contrast enhanced (Gadovist, Bayer, Germany) sequences were acquired. Additional sequences (e. g., liver sequences like diffusion-weighted or dynamic imaging) were acquired based on individual clinical settings. PET acquisition was performed in accordance with the EANM Research Ltd. (EARL) procedure guideline version 2.0 (EARL-2) for the PET/CT system, and PET acquisition on the PET/MRI system was performed in close adherence to the EARL-2 recommendations (24).

### Image analysis

All available images were evaluated by two senior nuclear medicine physicians and one senior radiologist, in accordance with the principles of consensus-based medical decision-making. In the absence of established tracer cut-offs, any suspect uptake above the background was considered and compared to morphological imaging. Morphologic suspect lesions with tracer uptake below the background were classified as negative on PET. Lesion classification was based on comparison with previous and/or follow-up imaging and the available morphological imaging findings to determine whether lesions were most consistent with metastatic disease. Following multidisciplinary tumor board discussion, selected patients were referred for biopsy or surgery for histopathological confirmation. Standardized uptake values (SUV) and metabolic [(^18^F)DOPA]/molecular [(^68^Ga)BED003] tumor volume (MTV) were obtained for all suspicious lesions. A total of four reference regions were measured in each imaging modality for each patient in order to perform a TBR analysis. Accordingly, a spheric volume of interest (VOI) was generated in the thoracic aorta for blood pool evaluation (1 cm^3^) as well as in the right upper lung lobe, the right liver lobe, and the right gluteus maximus muscle (each 3 cm^3^). The maximum TBR (TBR_max_) was calculated as a quotient of the lesion's maximum SUV (SUV_max_) and each reference mean SUV (SUV_mean_) (25).

### Statistical analysis

PET-, clinical- and demographic parameters are presented as total number, percentage, range, median and 95% confidence intervals (95% CI). The lesion detection rate of each PET modality is presented as paired binary dichotomous variables, which were compared using the exact McNemar test. Exact binomial confidence intervals were calculated using the Clopper-Pearson method. Quantitative PET parameters (SUV_max_, SUV_mean_, MTV and TBR_max_) were compared using linear mixed-effects models of log-transformed values, including tracer type as a fixed effect and patient- and lesion-specific random intercepts. Potential scanner effects (PET/CT vs. PET/MRI) were additionally assessed by including scanner type as a fixed effect. *P* values for multiple comparisons of quantitative PET parameters were adjusted using the Bonferroni and Benjamini-Hochberg method. To assess a potential influence of the interval between both examinations, sensitivity analyses were performed by excluding patients with scan intervals >42 days (6 weeks) and by including a tracer-by-scan-interval interaction term in the linear mixed-effects models. Statistical significance was assessed based on Bonferroni-adjusted *P* values. The null hypothesis was rejected if the *P* value was less than 0.05 (2-sided, if applicable). Given the exploratory, hypothesis-generating design and the rarity of advanced/recurrent MTC, no formal *a priori* sample-size or power calculation was performed. Statistical analysis was performed using SPSS Statistics version 28.0.1.1 (SPSS Inc., IBM, New York, USA) or R version 4.1.0 (R Foundation for Statistical Computing, Vienna, Austria).

## Results

### Patient-based analysis

A total of 8 patients who received [^18^F]DOPA- followed by [^68^Ga]BED003-PET were included in this retrospective study. Patient characteristics are detailed in Table 1. The median time between the two imaging modalities was 33 days (range: 7–56 d). Detailed time intervals are reported in Supplementary Table 1. [^18^F]DOPA- and [^68^Ga]BED003-positive lesions were observed in 7/8 (87.5%) and 6/8 (75%) patients, respectively. In patients #1, #2, #4, #6 and #7 [^68^Ga]BED003-PET detected more tracer-avid lesions than [^18^F]DOPA-PET. The same tracer-avid lesions were detected by both imaging modalities in patient #3. Patient #7 demonstrated left adrenal hyperplasia with increased [^18^F]DOPA-uptake but no [^68^Ga]BED003-uptake. Patient #8 demonstrated no [^18^F]DOPA-avid findings but exhibited symmetrical low [^68^Ga]BED003-uptake in mediastinal lymph nodes. Subsequent endobronchial ultrasonic-guided fine needle aspiration demonstrated the presence of chronic inflammation due to anthracosis without evidence of malignancy. In Patient #5 [^68^Ga]BED003-PET missed one supraclavicular lymph node metastasis, which revealed low [^18^F]DOPA-uptake. Figure 1 presents a comparison of tracer distribution among all patients, illustrated by maximum intensity projection.

**Figure 1:**
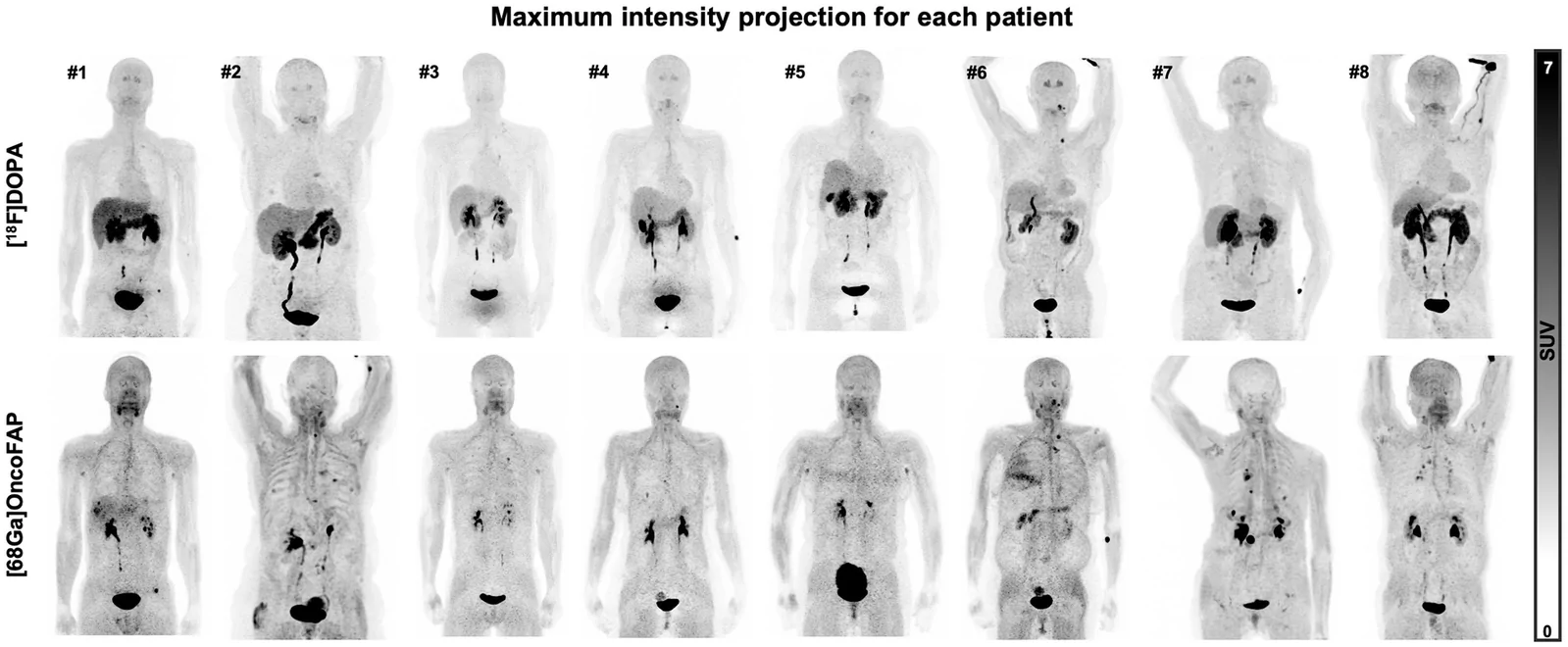
Comparison of both tracer distributions in each patient by maximum intensity projection. A comparison of tracer distribution by maximum intensity projection (SUV 0-7) was conducted for each patient and each PET modality. It was observed that the majority of MTC-related lesions exhibited a higher [^68^Ga]BED003 uptake. Moreover, a notable proportion of lesions exhibited no significant [^18^F]DOPA uptake yet exhibited notably high [^68^Ga]BED003 uptake.

### Lesion-based analysis

A total of 35 tracer-avid lesions were observed in both imaging modalities; of these 16/35 (45.7%; 95%CI, 28.8–63.4%) were [^18^F]DOPA-positive and 34/35 (97.1%; 95%CI, 85.1–99.9%) were [^68^Ga]BED003-positive. Of the aforementioned lesions, 15/35 (42.9%; 95%CI, 26.3–60.6%) were positive in both [^68^Ga]BED003- and [^18^F]DOPA-PET, while 19/35 (54.3%; 95%CI, 36.6–71.2%) were only [^68^Ga]BED003-positive and 1/35 (2.9%; 0.1–14.9%) was only [^18^F]DOPA-positive. Of these 35 tracer-avid lesions in comparison of [^18^F]DOPA-PET and [^68^Ga]BED003 avidity, 16/35 (45.7%) were classified as lymph node metastases (11/16 [68.8%] vs. 15/16 [93.8%]), 7/35 (20%) as bone metastases (4/7 [57.1%] vs. 7/7 [100%]), 6 (17.1%) as lung metastases (1/6 [16.7%] vs. 6/6 [100%]), 5/35 (14.3%) as liver metastases (0/5 [0%] vs. 5/5 [100%]) and 1/35 (2.9%) as a local recurrence (0/1 [0%] vs. 1/1 [100%]), respectively. Therefore, [^68^Ga]BED003-PET detected significantly more lesions than [^18^F]DOPA-PET (34 vs. 16; *P* < 0.001). After exclusion of the patient with the longest scan interval (#5, 56 days), the lesion-based detection results remained robust, with [^68^Ga]BED003 detecting 34 lesions compared with 15 lesions for [^18^F]DOPA (*P* < 0.001). The detailed lesional detection rates for both modalities are presented in Table 2.

**Table 2:** Lesion-based detection rate per patient with tumor markers.

Patient identification	Finding	[^18^F]DOPA	[^68^Ga]BED003	Calcitonin pg/mL	CEA ng/mL
#1	Left os ilium	+	+	33981	764
	T8 (Corpus vertebrae)	+	+		
	T10 (Corpus vertebrae)	+	+		
	Liver Seg. VII	–	+		
	Liver Seg. V (dorsal)	–	+		
	Liver Seg. V (ventral)	–	+		
#2	Local recurrence left	–	+	128	3.4
	Lung nodule lower lobe left	–	+		
	Hilary lymph node left	+	+		
#3	Supraclavicular lymph node left	+	+	2808	64.7
	Infraclavicular lymph node left	+	+		
	Hilary lymph node left	+	+		
#4	Cervical lymph node left, Level II	+	+	1344	40.7
	Cervical lymph node left, Level III	+	+		
	T8 (Corpus vertebrae)	–	+		
#5	Infraclavicular lymph node left	+	–	465	4.2
#6	Cervical lymph node left, Level IA	+	+	6150	N/A
	Cervical lymph node left, Level IB	–	+		
	Cervical lymph node left, Level II	+	+		
	Cervical lymph node left, Level II	+	+		
	Cervical lymph node left, Level III	–	+		
	Cervical lymph node left, Level III	–	+		
	Supraclavicular lymph node left	+	+		
	T2 (Corpus vertebrae)	–	+		
	T3 (Corpus vertebrae)	–	+		
	Left os ilium	+	+		
#7	Hilary lymph node left	–	+	274	51
	Hilary lymph node right	–	+		
	Lung nodule upper lobe left (dorsal)	+	+		
	Lung nodule upper lobe right (dorsal)	–	+		
	Lung nodule upper lobe right (ventral)	–	+		
	Lung nodule upper lobe left (ventral)	–	+		
	Lung nodule lower lobe left (dorsal)	–	+		
	Liver Seg. VII	–	+		
	Liver Seg. IV	–	+		
#8	No tumoral lesions were detected in both imaging modalities	−/−	−/−	1919	11

### Quantitative analysis

No significant scanner-related effects were observed for any of the quantitative PET parameters after adjustment for multiple comparisons; e. g., SUV_max_ showed a PET/MRI-to-PET/CT ratio of 0.88 (95%CI, 0.58–1.32; Bonferroni-adjusted *P* = 1.0). Detailed overview and analysis are presented in Supplementary Table 1. Longer scan intervals did not result in an increasing relative [^68^Ga]BED003-to-[^18^F]DOPA difference. Instead, the relative ratio decreased for several quantitative parameters, arguing against interval disease progression as an explanation for the higher [^68^Ga]BED003 uptake. Detailed results are presented in Supplementary Table 2.

The median SUV_max_ and SUV_mean_ of [^68^Ga]BED003 was significantly higher than that of [^18^F]DOPA (*n* = 35; SUV_max_: 6.62 vs. 2.1 and SUV_mean_: 3.9 vs. 1.5, both Bonferroni-adjusted *P* < 0.001). A total of 5/35 (14.3%) lesions in 3/7 (42.9%) patients demonstrated an [^68^Ga]BED003 SUVmax ≥10; however, none of the seven patients with metastatic lesions showed an SUVmax ≥10 in more than 50% of their lesions. The median MTV calculated of [^68^Ga]BED003 was significantly higher than that of [^18^F]DOPA (1.38 vs. 1.06 cm^3^, Bonferroni-adjusted *P* = 0.0033). All four calculated TBRs were significantly higher in [^68^Ga]BED003-PET than in [^18^F]DOPA-PET, e. g. median liver TBR_max_ with 9.83 vs. 0.94, adjusted *P* < 0.001 or median blood pool TBR_max_ with 6.02 vs. 2.23, Bonferroni-adjusted *P* < 0.001. The detailed statistical analysis is presented in Table 3. Case examples and PET findings are illustrated in Figures 2, 3. Detailed lesion-based and patient-based quantitative results and TBR_max_ analyses are presented in Supplementary Tables 3–5.

**Table 3:** Overview of the statistical comparison of quantitative PET parameters using linear mixed-effects models with adjustment for multiple comparisons.

Parameter	[^18^F]DOPA Median (Range)	[^68^Ga]BED003 Median (Range)	P value	BH-adjusted P value	Bonferroni-adjusted P value
SUV_max_	2.10 (0.70–10.50)	6.62 (2.50–34.20)	< 0.001	< 0.001	< 0.001
SUV_mean_	1.50 (0.50–6.10)	3.90 (1.50–24.10)	< 0.001	< 0.001	< 0.001
MTV (cm^3^)	1.06 (0.18–4.48)	1.38 (0.57–14.25)	< 0.001	< 0.001	0.0033
Liver TBR_max_	0.94 (0.30–4.38)	9.83 (3.73–57.00)	< 0.001	< 0.001	< 0.001
Muscle TBR_max_	3.22 (0.78–9.55)	5.41 (1.92–26.31)	< 0.001	< 0.001	< 0.001
Lung TBR_max_	8.50 (2.33–21.00)	10.78 (3.89–57.00)	< 0.001	< 0.001	< 0.001
Blood pool TBR_max_	2.23 (0.54–7.00)	6.02 (1.92–31.09)	< 0.001	< 0.001	< 0.001

**Figure 2:**
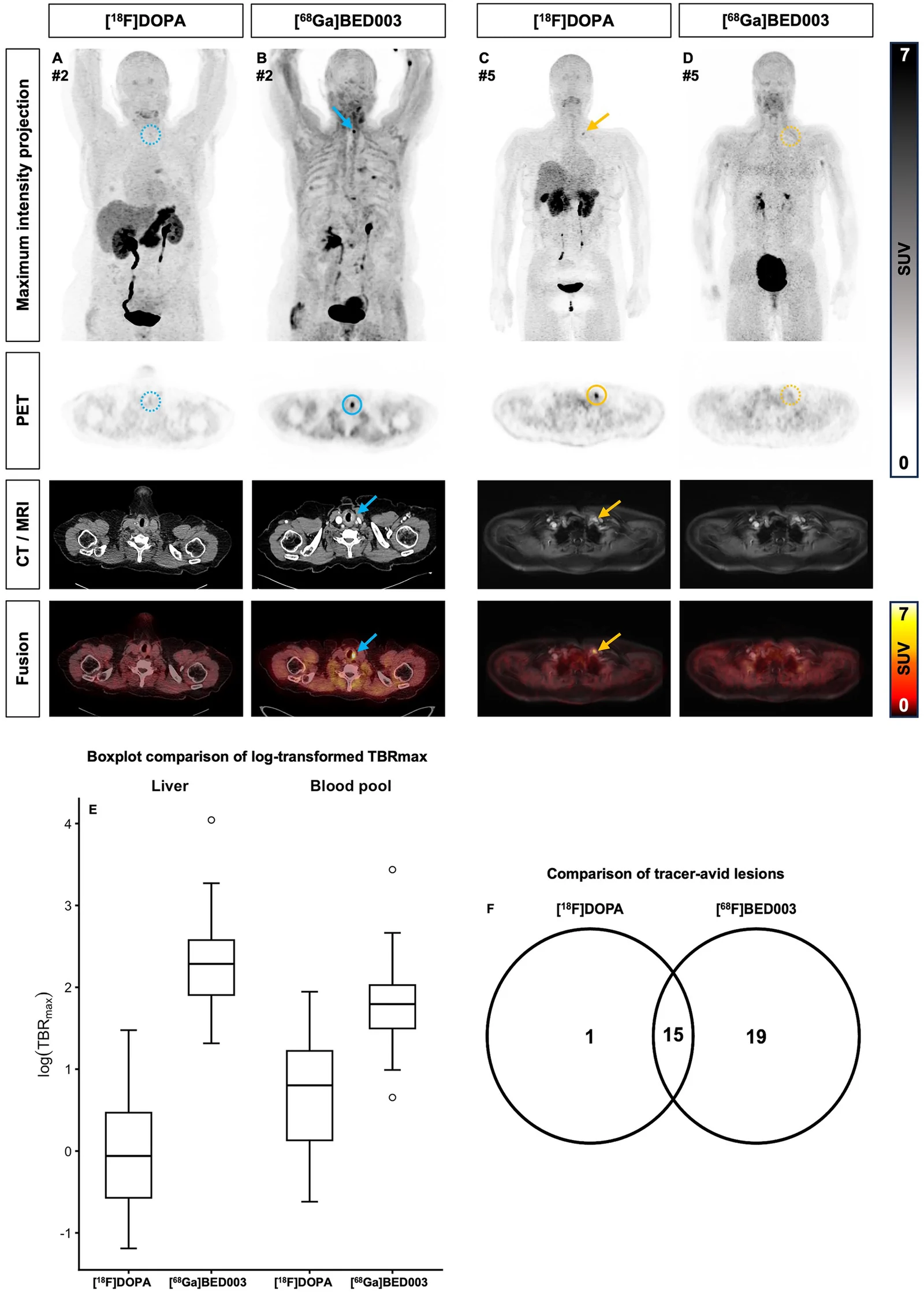
Case examples, quantitative and lesional comparison of PET findings. Patient #2 (**A/B**) revealed only a low-intense [^18^F]DOPA positive hilar lymph node metastasis (SUV_max_ 3.8), with a higher [^68^Ga]BED003 uptake (SUV_max_ 5.7). Furthermore, the presence of a high-intense [^68^Ga]BED003 positive local recurrence (SUV_max_ 9.9, blue arrow/circle) and lung metastasis of the left upper lobe (SUVmax 5.7) is evident, both without significant [^18^F]DOPA uptake (SUV_max_ 1.4 and 1.3, respectively). Patient #5 (**C/D**) demonstrated a suspicious left infraclavicular lymph node, characterized by pronounced contrast enhancement on MRI (orange arrow). This lymph node metastasis exhibited a slightly higher [^18^F]DOPA (SUV_max_ 3.5) than [^68^Ga]BED003 (SUV_max_ 2.5) uptake, thereby resulting in enhanced visibility on the [^18^F]DOPA-PET scan (orange circle). Following the excision of the aforementioned lymph node, a metastasis related to MTC was identified by histopathology. Boxplots **(E)** and analysis with linear mixed-effect models revealed a significant higher liver- and blood pool related TBR_max_ values for [^68^Ga]BED003 compared with [^18^F]DOPA. The median liver-related TBR_max_ was 9.83 (range, 3.73–57.0) vs. 0.94 (range, 0.3–4.38), while the median blood pool TBR_max_ was 6.02 (range, 1.92–31.09) vs. 2.23 (range: 0.54–7.0), both Bonferroni-adjusted *P* < 0.001, respectively. A comparison of tracer-avid lesions **(F)** revealed a significant higher number of lesions that were solely [^68^Ga]BED003 positive than solely [^18^F]DOPA positive (19 vs. 1, exact McNemar test *P* < 0.001), while 15 lesions were both tracer-avid.

**Figure 3:**
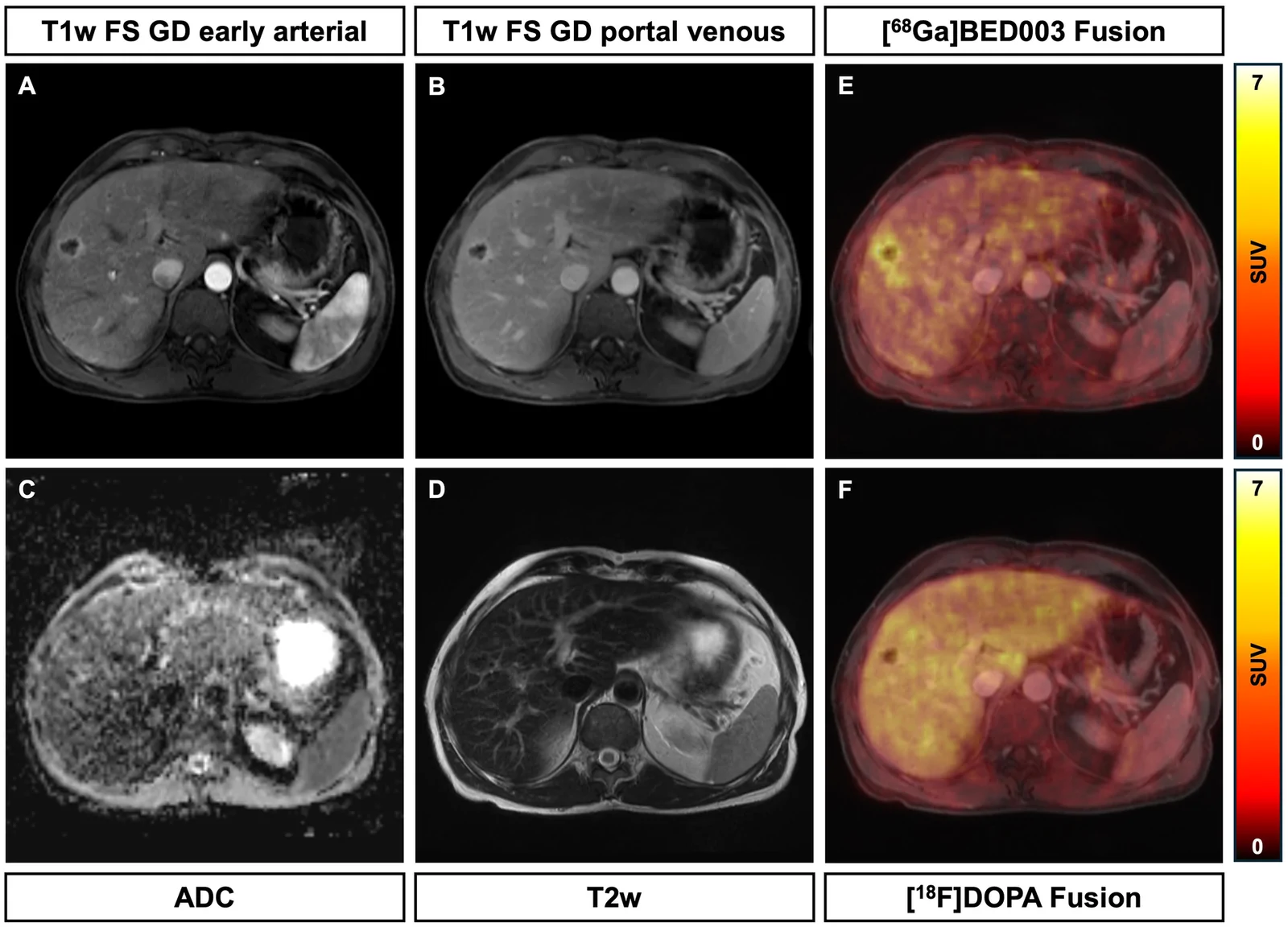
Case example of patient #1 with liver metastases and multiparametric PET-MRI. Multiparametric MRI of the liver revealed a liver lesion with early arterial hypervascularization and without central contrast agent wash-out in the portal venous phase (**A/B**). In diffusion weighted imaging the lesions presented as equivocal to surrounding tissue (no elevation of ADC-values) **(C)** In T2-weighted imaging the lesion was centrally isointense with peripheral hyperintensity **(D)** Furthermore, the lesion was found to be [^68^Ga]BED003-positive (SUVmax of 6.0; **E**) and [^18^F]DOPA-negative (SUVmax of 2.7 equal to liver background; **F**). A subsequent histopathological evaluation following a biopsy confirmed the presence of a MTC related liver metastasis.

### Histopathological confirmation and subsequent clinical management

Histopathological confirmation was obtained in 5/8 (62.5%) patients. In patient #1, excision biopsy confirmed an MTC liver metastasis that was [^18^F]DOPA-negative and [^68^Ga]BED003-positive, whereas adrenalectomy demonstrated a pheochromocytoma that was [^18^F]DOPA-positive and [^68^Ga]BED003-negative. In patient #2, endobronchial ultrasound-guided transbronchial needle aspiration confirmed a local MTC recurrence and a left hilar lymph node metastasis, whereas the local recurrence was [^18^F]DOPA-negative. In patient #5, lymphadenectomy confirmed an [^18^F]DOPA-positive/[^68^Ga]BED003-negative infraclavicular MTC lymph node metastasis. In patient #7, liver segment resection confirmed two hepatic MTC metastases, both [^18^F]DOPA-negative and [^68^Ga]BED003-positive. In patient #8, biopsy of [^18^F]DOPA-negative/[^68^Ga]BED003-positive hilar lymph nodes demonstrated inflammation and anthracosis without malignancy. Detailed histopathological findings are presented in Table 4.

**Table 4:** Histopathological findings and corresponding [^18^F]dOPA and [^68^Ga]BED003-PET results.

Patient	Biopsy/Surgery	Histology	[^18^F]DOPA SUV_max_	DOPA	[^68^Ga]BED003 SUV_max_	BED003
#1	Excision biopsy	Liver Segment V: MTC metastasis	2.7	–	6.0	+
	Adrenalectomy	Left adrenal gland: Pheochromocytoma	7.4	+	2.1	–
#2	Endobronchial ultrasound-guided transbronchial needle aspiration	Tracheal lesion: Local MTC recurrence	2.7	–	9.9	+
		Left hilar lymph node: MTC metastasis	3.8	+	5.7	+
#3	N/A	N/A	N/A	N/A	N/A	N/A
#4	N/A	N/A	N/A	N/A	N/A	N/A
#5	Lymphadenectomy	Left infraclavicular lymph node: MTC metastasis	3.5	+	2.5	–
#6	N/A	N/A	N/A	N/A	N/A	N/A
#7	Atypical segmental liver resection	Liver Segment IV: MTC metastasis	2.9	–	34.2	+
		Liver Segment VII: MTC metastasis	2.3	–	15.8	+
#8	Endobronchial ultrasound-guided transbronchial needle aspiration	Hilar lymph nodes: Inflammation/anthracosis	2.4	–	Up to 5.1	+

Subsequent management included adrenalectomy in patient #1, confirming a pheochromocytoma in the context of MEN2A syndrome, while biopsy of the [^18^F]DOPA-negative/[^68^Ga]BED003-positive liver lesion excluded pheochromocytoma metastasis and confirmed MTC metastasis. Patient #1 was subsequently managed with active surveillance and imaging and tumor-marker follow-up. Patient #2 underwent stereotactic radiotherapy of the local recurrence, left hilar lymph node metastasis, and a pulmonary metastasis, followed by a decrease in calcitonin from 128 to 99.1 pg/mL and CEA from 3.4 to 2.0 ng/mL. Patient #5 underwent left-sided lymphadenectomy; calcitonin and CEA subsequently increased further in association with new metastases on follow-up [^18^F]DOPA-PET. Patient #6 started osteoprotective treatment with denosumab. Patient #7 underwent liver segment resection followed by selpercatinib, with a decrease in calcitonin from 274 to 5.5 pg/mL and CEA from 51 to 22.3 ng/mL. Patients #3, #4, and #8 were managed with active surveillance. Overall, [^68^Ga]BED003-PET findings contributed to subsequent management decisions in three patients (#2, #6, and #7), while in patient #1 they prompted additional diagnostic clarification.

## Discussion

Although several imaging modalities are available, localization and staging of recurrent or metastatic MTC remain challenging (1, 2, 4). In the present study, eight patients underwent [^18^F]DOPA-PET followed by [^68^Ga]BED003-PET. Despite the limited cohort size, which reflects the rarity of advanced MTC, [^68^Ga]BED003-PET detected more lesions than [^18^F]DOPA-PET and showed higher lesional uptake, MTV, and TBRs. These findings support FAP-targeted PET as a promising complementary imaging approach in MTC.

[^18^F]DOPA-PET is an established reference standard for recurrent MTC, but detection depends on calcitonin levels, tumour burden, lesion size, and lesion location (4–6, 9). SSTR-directed PET is often used as a complementary modality, particularly because it may identify candidates for PRRT (4). However, SSTR expression in MTC is heterogeneous, and comparative studies indicate that [^18^F]DOPA-PET and SSTR-PET are complementary, with [^18^F]DOPA often showing equal or superior lesion detection (9–11). In this context, our results are consistent with recent FAP-targeted imaging data: Ballal et al. reported superior lesion detection with [^68^Ga]Ga-DOTA.SA.FAPi compared with [^68^Ga]Ga-DOTANOC PET/CT (15). Hescheler et al. recently reported a head-to-head comparison of [^68^Ga]FAPI and [^18^F]DOPA PET/CT in MTC, demonstrating higher lesion detection rates and higher TBRs for FAPI-PET, particularly for lymph node, liver, and lung metastases (18). The present study extends these observations to [^68^Ga]BED003 PET, a high-affinity tracer derived from the OncoFAP platform (12–14).

The most relevant differences were observed in liver and lung lesions. In patients #1 and #7, MRI-suspicious liver lesions showed no relevant [^18^F]DOPA uptake above liver background but intense focal [^68^Ga]BED003 uptake. This is clinically plausible, as liver staging in MTC often benefits from complementary MRI, particularly for small or [^18^F]DOPA-negative metastases (26). Physiological hepatic and biliary [^18^F]DOPA activity may reduce lesion conspicuity, whereas FAPI tracers usually show low physiological liver background and high TBRs (13, 21). Similar limitations may also apply to SSTR-PET because of physiological liver uptake (27). However, FAP uptake is not tumour specific (13): in patient #4, liver lesions with intense [^68^Ga]BED003 uptake but no [^18^F]DOPA uptake were classified as most likely haemangiomas based on typical MRI findings and long-term stability. This is important because hepatic fibrosis, inflammation, post-treatment changes, and benign liver tumours may be FAP-positive and can represent relevant pitfalls (13, 28, 29).

False-positive FAP uptake was also observed in patient #8, who showed symmetrical [^68^Ga]BED003-positive mediastinal lymphadenopathy caused by histologically proven anthracosis. Despite elevated calcitonin levels, no tracer-avid MTC lesions were detected in either modality. This case illustrates that inflammatory or fibrotic lymph node changes may be FAP-positive and that a negative FAPI-PET scan cannot reliably exclude MTC-related disease in all patients. Similar caution is required in inflammatory fibroblast activation, including sarcoidosis (30, 31).

In patients #2 and #7, several pulmonary metastases were [^18^F]DOPA-negative but intensely [^68^Ga]BED003-positive. Many of these lesions were small on CT, which is typical for pulmonary MTC metastases and makes them susceptible to respiratory motion and partial-volume effects (32). Given the relatively small metabolic tumour volumes observed in the present cohort, partial-volume effects may have led to an underestimation of lesional uptake, particularly in small lesions. This may be especially relevant for [^18^F]DOPA, where lower tumour-to-background contrast may additionally reduce lesion conspicuity. High [^68^Ga]BED003 uptake and low lung background may therefore improve visualization of low-volume pulmonary disease. In patients #3–#6, most cervical lymph node metastases were visible in both modalities, while bone metastases in patients #1, #4, and #6 were more conspicuous or exclusively visible on [^68^Ga]BED003-PET. This organ-specific pattern is in line with the recent FAPI-vs-DOPA comparison, which also reported relevant advantages of FAPI-PET for several metastatic sites (18).

The present study also demonstrates tracer-specific pitfalls of [^18^F]DOPA-PET. In patients with MEN2 syndrome or suspected hereditary disease, [^18^F]DOPA uptake in pheochromocytoma, paraganglioma, adrenal medulla, or adrenal hyperplasia may complicate interpretation (33, 34). Accordingly, patient #1 had a histologically proven pheochromocytoma and patient #7 showed adrenal hyperplasia, both with high [^18^F]DOPA uptake but without corresponding [^68^Ga]BED003 uptake. In addition, acquisition timing may influence [^18^F]DOPA performance. Early imaging protocols have been reported to be safe and diagnostically relevant, or slightly superior, in recurrent MTC (35, 36). Since no early [^18^F]DOPA acquisition was performed in the present study, future comparisons should include standardized early and late [^18^F]DOPA protocols.

Beyond diagnostic imaging, FAP-targeted PET may have theranostic implications. SSTR-PET is already used as a gatekeeper for PRRT with [^177^Lu]DOTATATE or related SSTR ligands in selected patients with metastatic MTC, but PRRT is not established as routine standard therapy because SSTR expression and treatment evidence remain heterogeneous (11, 37). FAP-targeted radioligand therapy may represent an additional strategy for patients with sufficient FAP expression. Initial experience with [^90^Y]FAPI-46 showed feasibility but suggested that high tumour uptake is required, with SUVmax ≥10 in more than 50% of lesions used as a practical selection criterion (38, 39). In the present cohort, only 5/35 (14.3%) lesions reached an [^68^Ga]BED003 SUV_max_ ≥10, occurring in 3/7 (42.9%) patients with metastatic lesions. However, none of these patients demonstrated an SUV_max_ ≥10 in more than 50% of their lesions and therefore none fulfilled the previously proposed patient-level uptake criterion (38, 39). The median lesional SUVmax was 6.62. However, partial-volume effects in small lesions may have underestimated [^68^Ga]BED003 SUVmax and should be considered when applying uptake-based theranostic thresholds. Overall, these findings suggest that uptake may be insufficient for many currently available short-retention FAPI therapeutic compounds, restricting their potential use to selected cases. Improved biochemical properties of novel compounds may promote more durable, target-specific binding, which could potentially broaden the clinical applications of this theranostic approach.

This study is limited by its single-center retrospective design, small cohort size, and the lack of histopathological confirmation for all lesions. Lesion classification relied on a composite reference standard including CT, MRI, follow-up imaging, and clinical interpretation; therefore, incorporation bias and observer bias cannot be fully excluded. In addition, [^18^F]DOPA-PET was always performed before [^68^Ga]BED003-PET, so interval disease progression may have influenced the comparison, particularly in patients with longer interscan intervals. However, sensitivity analyses excluding the patient with the longest interval and explicitly modelling the scan interval yielded consistent results and did not indicate an increasing relative [^68^Ga]BED003-to-[^18^F]DOPA-ratio difference with longer intervals. Importantly, the cohort was clinically selected for additional [^68^Ga]BED003 imaging after [^18^F]DOPA PET had been considered insufficient in the context of biochemical or imaging findings. This introduces spectrum and selection bias and may have contributed to the magnitude of the observed difference in lesion detection rates, limiting generalizability to an unselected recurrent MTC population. Four of eight patients had previously been included in our multicancer [^68^Ga]BED003 analysis (21), although the present paired head-to-head comparison with [^18^F]DOPA and lesion-based analyses were not previously reported. Finally, no early [^18^F]DOPA acquisition, FAP immunohistochemistry, or lesion-based dosimetry was available. Despite these limitations, the present findings suggest that [^68^Ga]BED003 may represent a useful complementary imaging option, particularly in patients in whom [^18^F]DOPA PET provides insufficient diagnostic information.

## Conclusion

In this small MTC cohort, [^68^Ga]BED003-PET demonstrated higher lesion detection and higher TBR contrast, adding additional clinical value in patients with inconclusive findings in [^18^F]DOPA-PET. FAP-targeted PET may therefore represent a promising complementary imaging approach in recurrent or metastatic MTC.

## Data Availability

The original contributions presented in the study are included in the article/Supplementary Material, further inquiries can be directed to the corresponding author.
