# Supplementary material for: FAP-targeted [68Ga]BED003-PET compared with [18F]DOPA-PET in patients with recurrent medullary thyroid carcinoma
Source: Front Nucl Med. 2026 Oct 21;6:1949036. doi: 10.3389/fnume.2026.1949036 (PMC13731261; doi:10.3389/fnume.2026.1949036)
Supplement: Supplementary file 1 [file Table1.docx]

**SUPPLEMENTS**

**Table 1S:** Patient-based scanner overview and statistical assessment of scanner-related effects on quantitative PET parameters using linear mixed-effects models

| **Patient** | **[^18^F]DOPA** | **[^68^Ga]BED003** | **Days between both PET scans (days)** |
| --- | --- | --- | --- |
| **#1** | PET/MRI | PET/MRI | 39 |
| **#2** | PET/CT | PET/CT | 14 |
| **#3** | PET/MRI | PET/MRI | 33 |
| **#4** | PET/MRI | PET/MRI | 38 |
| **#5** | PET/MRI | PET/MRI | 56 |
| **#6** | PET/CT | PET/MRI | 33 |
| **#7** | PET/CT | PET/CT | 23 |
| **#8** | PET/CT | PET/CT | 7 |

| **Parameter** | **PET/MRI-to-PET/CT ratio** | **95%CI** | **P value** | **Benjamini-Hochberg adjusted P value** | **Bonferroni-adjusted P value** |
| --- | --- | --- | --- | --- | --- |
| **SUVmax** | 0.88 | 0.58–1.32 | 0.517 | 0.660 | 1.000 |
| **SUVmean** | 0.84 | 0.55–1.27 | 0.380 | 0.660 | 1.000 |
| **MTV** | 0.74 | 0.44–1.25 | 0.247 | 0.660 | 1.000 |
| **Liver TBRmax** | 0.84 | 0.55–1.29 | 0.404 | 0.660 | 1.000 |
| **Muscle TBRmax** | 1.01 | 0.64–1.60 | 0.962 | 0.962 | 1.000 |
| **Lung TBRmax** | 0.88 | 0.57–1.38 | 0.566 | 0.660 | 1.000 |
| **Blood pool TBRmax** | 1.17 | 0.76–1.81 | 0.410 | 0.660 | 1.000 |

**Table 2S:** Sensitivity analysis of the effect of scan interval on quantitative PET parameters

| **Parameter** | **[68Ga]BED003/[18F]DOPA ratio change per 10 days** | **95% CI** | **Bonferroni-adjusted P value** |
| --- | --- | --- | --- |
| SUVmax | 0.63 | 0.48 – 0.83 | 0.012 |
| SUVmean | 0.63 | 0.49 – 0.83 | 0.010 |
| MTV | 1.21 | 0.85 – 1.71 | 1.000 |
| Liver TBRmax | 0.58 | 0.44 – 0.77 | 0.003 |
| Muscle TBRmax | 0.63 | 0.47 – 0.84 | 0.017 |
| Lung TBRmax | 0.72 | 0.54 – 0.96 | 0.191 |
| Blood pool TBRmax | 0.58 | 0.43 – 0.77 | 0.003 |

**Table 3S:** Quantitative results of standard PET parameters

|  |  | **[^18^F]DOPA** | | | **[^68^Ga]BED003** | | |
| --- | --- | --- | --- | --- | --- | --- | --- |
| **Patients/Lesions** | | **SUV_MAX_** | **SUV_MEAN_** | **MTV (cm^3^)** | **SUV_MAX_** | **SUV_MEAN_** | **MTV (cm^3^)** |
| **#1** | **L_01** | 6.1 | 3.6 | 2.44 | 10.8 | 6.3 | 4.32 |
|  | **L_02** | 2.1 | 1.4 | 0.18 | 4.1 | 2.6 | 1.24 |
|  | **L_03** | 1.9 | 1.3 | 1.38 | 4.9 | 2.8 | 1.98 |
|  | **L_04** | 3.5 | 2.6 | 1.41 | 7.4 | 4.1 | 2.40 |
|  | **L_05** | 2.7 | 2.2 | 0.28 | 6.0 | 3.4 | 1.80 |
|  | **L_06** | 3.2 | 2.4 | 0.34 | 6.8 | 4.0 | 14.25 |
| **#2** | **L_07** | 1.4 | 1.2 | 0.33 | 9.9 | 5.6 | 0.96 |
|  | **L_08** | 1.3 | 0.7 | 3.95 | 6.8 | 3.9 | 1.92 |
|  | **L_09** | 3.8 | 2.3 | 4.48 | 5.7 | 3.2 | 6.57 |
| **#3** | **L_10** | 1.7 | 1.0 | 0.69 | 4.3 | 2.3 | 2.30 |
|  | **L_11** | 1.9 | 1.2 | 1.24 | 3.3 | 1.8 | 1.84 |
|  | **L_12** | 2.6 | 1.5 | 2.90 | 6.3 | 3.6 | 2.1 |
| **#4** | **L_13** | 3.3 | 1.9 | 0.85 | 9.1 | 6.4 | 0.64 |
|  | **L_14** | 5.2 | 3.2 | 0.35 | 5.9 | 3.4 | 0.74 |
|  | **L_15** | 1.2 | 1.0 | 0.28 | 6.62 | 4.2 | 0.57 |
| **#5** | **L_16** | 3.5 | 2.1 | 1.06 | 2.5 | 1.5 | 1.00 |
| **#6** | **L_17** | 9.5 | 5.6 | 1.96 | 9.2 | 6.5 | 1.38 |
|  | **L_18** | 1.8 | 1.3 | 1.00 | 8.7 | 5.5 | 1.13 |
|  | **L_19** | 6.1 | 4.2 | 0.56 | 9.7 | 5.9 | 1.24 |
|  | **L_20** | 4.3 | 2.8 | 0.33 | 5.7 | 3.2 | 1.24 |
|  | **L_21** | 1.8 | 1.5 | 0.43 | 7.6 | 4.6 | 0.64 |
|  | **L_22** | 1.5 | 1.3 | 0.23 | 6.1 | 3.4 | 1.2 |
|  | **L_23** | 10.5 | 6.1 | 1.33 | 11.1 | 6.7 | 2.48 |
|  | **L_24** | 1.9 | 1.5 | 1.33 | 5.4 | 3.1 | 0.99 |
|  | **L_25** | 1.2 | 0.9 | 0.43 | 8.1 | 4.8 | 0.64 |
|  | **L_26** | 6.7 | 3.9 | 0.70 | 3.5 | 1.9 | 0.60 |
| **#7** | **L_27** | 0.8 | 0.5 | 1.34 | 11.1 | 5.9 | 4.38 |
|  | **L_28** | 1.6 | 1.4 | 2.34 | 3.1 | 1.9 | 1.89 |
|  | **L_29** | 2.8 | 1.7 | 2.22 | 4.1 | 2.6 | 1.65 |
|  | **L_30** | 1.1 | 0.7 | 1.19 | 7.1 | 4.1 | 1.16 |
|  | **L_31** | 0.8 | 0.5 | 1.39 | 8.4 | 4.8 | 0.83 |
|  | **L_32** | 0.8 | 0.6 | 1.06 | 3.1 | 1.7 | 3.28 |
|  | **L_33** | 0.7 | 0.5 | 1.49 | 5.9 | 3.3 | 2.99 |
|  | **L_34** | 2.3 | 1.9 | 0.76 | 15.8 | 9.2 | 0.96 |
|  | **L_35** | 2.9 | 1.9 | 0.89 | 34.2 | 24.1 | 3.98 |

**Table 4S:** Overview of median quantitative PET values per patient

|  |  | **Median [^18^F]DOPA (range)** | | | **Median [^68^Ga]BED003 (range)** | | |
| --- | --- | --- | --- | --- | --- | --- | --- |
| **Patient** | | **SUV_MAX_** | **SUV_MEAN_** | **MTV (cm^3^)** | **SUV_MAX_** | **SUV_MEAN_** | **MTV (cm^3^)** |
| **#1** | | 2.95  (1.90–6.10) | 2.30  (1.30–3.60) | 0.86  (0.18–2.44) | 6.40  (4.10–10.80) | 3.70  (2.60–6.30) | 2.19  (1.24–14.25) |
| **#2** | | 1.40  (1.30–3.80) | 1.20  (0.70–2.30) | 3.95  (0.33–4.48) | 6.80  (5.70–9.90) | 3.90  (3.20–5.60) | 1.92  (0.96–6.57) |
| **#3** | | 1.90  (1.70–2.60) | 1.20  (1.00–1.50) | 1.24  (0.69–2.90) | 4.30  (3.30–6.30) | 2.30  (1.80–3.60) | 2.10  (1.84–2.30) |
| **#4** | | 3.30  (1.20–5.20) | 1.90  (1.00–3.20) | 0.35  (0.28–0.85) | 6.62  (5.90–9.10) | 4.20  (3.40–6.40) | 0.64  (0.57–0.74) |
| **#5** | | 3.50  (N/A) | 2.10  (N/A) | 1.06  (N/A) | 2.50  (N/A) | 1.50  (N/A) | 1.00  (N/A) |
| **#6** | | 3.10  (1.20–10.50) | 2.15  (0.90–6.10) | 0.63  (0.23–1.96) | 7.85  (3.50–11.10) | 4.70  (1.90–6.70) | 1.17  (0.60–2.48) |
| **#7** | | 1.10  (0.70–2.90) | 0.70  (0.50–1.90) | 1.34  (0.76–2.34) | 7.10  (3.10–34.20) | 4.10  (1.70–24.10) | 1.89  (0.83–4.38) |

**Table 5S:** Quantitative analysis of TBR_MAX_

|  |  | **[^18^F]DOPA TBR_MAX_** | | | | **[^68^Ga]OncoFAP TBR_MAX_** | | | |
| --- | --- | --- | --- | --- | --- | --- | --- | --- | --- |
| **Patients/Lesions** | | **Liver** | **Muscle** | **Lung** | **Blood** | **Liver** | **Muscle** | **Lung** | **Blood** |
| **#1** | **L_01** | 1.8 | 8.7 | 20.3 | 6.8 | 9.8 | 13.5 | 36.0 | 12.0 |
|  | **L_02** | 0.6 | 3.0 | 7.0 | 2.3 | 3.7 | 5.1 | 13.7 | 4.6 |
|  | **L_03** | 0.6 | 2.7 | 6.3 | 2.1 | 4.5 | 6.1 | 16.3 | 5.4 |
|  | **L_04** | 1.0 | 5.0 | 11.7 | 3.9 | 6.7 | 9.3 | 24.7 | 8.2 |
|  | **L_05** | 0.8 | 3.9 | 9.0 | 3.0 | 5.5 | 7.5 | 20.0 | 6.7 |
|  | **L_06** | 0.9 | 4.6 | 10.7 | 3.6 | 6.2 | 8.5 | 22.7 | 7.6 |
| **#2** | **L_07** | 0.6 | 1.8 | 4.7 | 1.2 | 16.5 | 8.3 | 19.8 | 9.0 |
|  | **L_08** | 0.5 | 1.6 | 4.3 | 1.1 | 11.3 | 5.7 | 13.6 | 6.2 |
|  | **L_09** | 1.5 | 4.8 | 12.7 | 3.2 | 9.5 | 4.8 | 11.4 | 5.2 |
| **#3** | **L_10** | 1.1 | 3.4 | 8.5 | 2.1 | 7.2 | 5.4 | 7.2 | 3.9 |
|  | **L_11** | 1.2 | 3.8 | 9.5 | 2.4 | 5.5 | 4.1 | 5.5 | 3.0 |
|  | **L_12** | 1.6 | 5.2 | 13.0 | 3.3 | 10.5 | 7.9 | 10.5 | 5.7 |
| **#4** | **L_13** | 1.6 | 4.7 | 11.0 | 3.3 | 18.2 | 8.3 | 15.2 | 8.3 |
|  | **L_14** | 2.5 | 7.4 | 17.3 | 5.2 | 11.8 | 5.4 | 9.8 | 5.4 |
|  | **L_15** | 0.6 | 1.7 | 4.0 | 1.2 | 13.2 | 6.0 | 11.0 | 6.0 |
| **#5** | **L_16** | 1.8 | 5.8 | 11.7 | 3.5 | 4.2 | 1.9 | 6.3 | 1.9 |
| **#6** | **L_17** | 4.0 | 8.6 | 19.0 | 6.3 | 13.1 | 5.4 | 10.2 | 7.1 |
|  | **L_18** | 0.8 | 1.6 | 3.6 | 1.2 | 12.4 | 5.1 | 9.7 | 6.7 |
|  | **L_19** | 2.5 | 5.5 | 12.2 | 4.1 | 13.9 | 5.7 | 10.8 | 7.5 |
|  | **L_20** | 1.8 | 3.9 | 8.6 | 2.9 | 8.1 | 3.4 | 6.3 | 4.4 |
|  | **L_21** | 0.8 | 1.6 | 3.6 | 1.2 | 10.9 | 4.5 | 8.4 | 5.8 |
|  | **L_22** | 0.6 | 1.4 | 3.0 | 1.0 | 8.7 | 3.6 | 6.8 | 4.7 |
|  | **L_23** | 4.4 | 9.5 | 21.0 | 7.0 | 15.9 | 6.5 | 12.3 | 8.5 |
|  | **L_24** | 0.8 | 1.7 | 3.8 | 1.3 | 7.7 | 3.2 | 6.0 | 4.2 |
|  | **L_25** | 0.5 | 1.1 | 2.4 | 0.8 | 11.6 | 4.8 | 9.0 | 6.2 |
|  | **L_26** | 2.8 | 6.1 | 13.4 | 4.5 | 5.0 | 2.1 | 3.9 | 2.7 |
| **#7** | **L_27** | 0.3 | 0.9 | 2.7 | 0.6 | 18.5 | 8.5 | 18.5 | 10.1 |
|  | **L_28** | 0.7 | 1.8 | 5.3 | 1.2 | 5.2 | 2.4 | 5.2 | 2.8 |
|  | **L_29** | 0.4 | 1.0 | 3.0 | 0.7 | 6.8 | 3.2 | 6.8 | 3.7 |
|  | **L_30** | 0.5 | 1.2 | 3.7 | 0.8 | 11.8 | 5.5 | 11.8 | 6.5 |
|  | **L_31** | 0.3 | 0.9 | 2.7 | 0.6 | 14.0 | 6.5 | 14.0 | 7.6 |
|  | **L_32** | 0.3 | 0.9 | 2.7 | 0.6 | 5.2 | 2.4 | 5.2 | 2.8 |
|  | **L_33** | 0.3 | 0.8 | 2.3 | 0.5 | 9.8 | 4.5 | 9.8 | 5.4 |
|  | **L_34** | 1.0 | 2.6 | 7.7 | 1.8 | 26.3 | 12.2 | 26.3 | 14.4 |
|  | **L_35** | 1.3 | 3.2 | 9.7 | 2.2 | 57.0 | 26.3 | 57.0 | 31.1 |
